# An Explainable Classification Method Based on Complex Scaling in Histopathology Images for Lung and Colon Cancer

**DOI:** 10.3390/diagnostics13091594

**Published:** 2023-04-29

**Authors:** Sudhakar Tummala, Seifedine Kadry, Ahmed Nadeem, Hafiz Tayyab Rauf, Nadia Gul

**Affiliations:** 1 Department of Electronics and Communication Engineering, School of Engineering and Sciences, SRM University-AP, Amaravati 522240, Andhra Pradesh, India; 2 Department of Applied Data Science, Noroff University College, 4612 Kristiansand, Norway; 3 Artificial Intelligence Research Center (AIRC), Ajman University, Ajman 346, United Arab Emirates; 4 Department of Electrical and Computer Engineering, Lebanese American University, Byblos P.O. Box 36, Lebanon; 5 Department of Pharmacology & Toxicology, College of Pharmacy, King Saud University, P.O. Box 2455, Riyadh 11451, Saudi Arabia; 6 Centre for Smart Systems, AI and Cybersecurity, Staffordshire University, Stoke-on-Trent ST4 2DE, UK; 7 Wah Medical College affiliated with POF Hospital, Wah Cantt 47040, Pakistan; mrsnadiagul@gmail.com

**Keywords:** lung cancer, colon cancer, *EffcientNetV2*, explainability, histopathology

## Abstract

Lung and colon cancers are among the leading causes of human mortality and morbidity. Early diagnostic work up of these diseases include radiography, ultrasound, magnetic resonance imaging, and computed tomography. Certain blood tumor markers for carcinoma lung and colon also aid in the diagnosis. Despite the lab and diagnostic imaging, histopathology remains the gold standard, which provides cell-level images of tissue under examination. To read these images, a histopathologist spends a large amount of time. Furthermore, using conventional diagnostic methods involve high-end equipment as well. This leads to limited number of patients getting final diagnosis and early treatment. In addition, there are chances of inter-observer errors. In recent years, deep learning has shown promising results in the medical field. This has helped in early diagnosis and treatment according to severity of disease. With the help of *EffcientNetV2* models that have been cross-validated and tested fivefold, we propose an automated method for detecting lung (lung adenocarcinoma, lung benign, and lung squamous cell carcinoma) and colon (colon adenocarcinoma and colon benign) cancer subtypes from LC25000 histopathology images. A state-of-the-art deep learning architecture based on the principles of compound scaling and progressive learning, *EffcientNetV2* large, medium, and small models. An accuracy of 99.97%, AUC of 99.99%, F1-score of 99.97%, balanced accuracy of 99.97%, and Matthew’s correlation coefficient of 99.96% were obtained on the test set using the *EffcientNetV2*-L model for the 5-class classification of lung and colon cancers, outperforming the existing methods. Using gradCAM, we created visual saliency maps to precisely locate the vital regions in the histopathology images from the test set where the models put more attention during cancer subtype predictions. This visual saliency maps may potentially assist pathologists to design better treatment strategies. Therefore, it is possible to use the proposed pipeline in clinical settings for fully automated lung and colon cancer detection from histopathology images with explainability.

## 1. Introduction

According to the World Health Organization, cancer is the leading cause of mortality worldwide, and by 2040, the global cancer burden is expected to be 28.4 million cases, a 47% increase from 2020 [1,2]. Lung and colorectal (both colon and rectum) cancers are the more prevalent types worldwide, after breast cancer, with incidence rates of 11.4% and 10%, respectively, in 2020 [2]. Although low, there is a chance of synchronous occurrence between lung and colon cancers [3]. In addition, lung and colorectal cancers exhibit the top two mortality rates of 18% and 9.4%, respectively, among all cancers [2]. Therefore, a more accurate diagnosis of these cancer subtypes is necessary to explore the treatment options in the initial stages of the disease. The non-invasive methods for diagnosis include radiography and computed tomography (CT) imaging for lung cancer and flexible sigmoidoscopy, and CT colonoscopy for colon cancer. However, reliable subtyping of these cancers may not be possible using non-invasive means always, and minimally invasive procedures, such as histopathology, would be required for precise disease identification and improved quality of treatment. In addition, the manual grading of histopathological images may be tiresome to pathologists. Moreover, accurate grading of the lung and colon cancer subtypes requires trained pathologists, and manual grading could be error prone. Hence, automated image processing methods for cancer lung and colon cancer subtype screening are warranted to reduce the burden on pathologists.

Deep learning (DL) is a branch of machine learning (ML) that eliminates the need for manual feature engineering, and convolutional neural network (CNN) based DL models provide hierarchical feature maps for better representation of input images. In recent years, various state-of-the-art CNN-based DL frameworks, including the AlexNet [4], VGG Nets [5], GoogLeNet [6], Residual Nets [7], DenseNets [8], EfficientNets [9,10], and, lately, multi-head self-attention based vision transformer (ViT) [11,12] architectures were invented for various vision tasks, including classification. Although massive data would be required to train these large DL models from scratch, transfer learning (TL) helps to adapt the large pre-trained models for downstream tasks. Thus, TL reduces the need for massive data for training, which is scarce in specific fields, such as medicine. DL and TL have been performing a vital role in healthcare in building automated diagnostic systems using medical images that include histopathological images, retina images, radiographs, computed tomography images, magnetic resonance images, etc. These automated systems are primarily used for classification tasks and also assist clinical practitioners in situations of rapid data acquisition and automated quality checking [13,14,15,16,17]. *EffcientNetV2* is a recent DL architecture that was developed based on progressive learning with a combination of compound scaling and neural architecture search (NAS) to improve both the training speed and parameter efficiency [10], and it outperformed several existing state-of-the-art DL models, including ViT variants in image classification task on the ImageNet and other datasets.

In general, DL methods are similar to black box architectures. Therefore, it is often required to ensure that these DL models focus on the most relevant regions in the input image during target class prediction. Several methods exist in the literature to visualize most activated areas when a DL model predicts the class of a specific image to add explainability to the model. A few of these methods include class activation mapping (CAM) [18], gradCAM [19], and gradCAM++ [20]. In this study, we considered gradCAM for creating visual saliency maps for *EffcientNetV2* predictions.

Hence, the contributions of the present work are:

i. A fully automated framework for the five-class diagnosis of most occurring lung and colon cancer subtypes is proposed using *EffcientNetV2*-large (*L*), medium (*M*), and small (*S*) models based on histopathology images.
ii. These existing pretrained models are finetuned and tested using a large, openly available lung and colon cancer histopathology image dataset called LC25000.
iii. Visual saliency maps are provided using the gradCAM method to understand the model decisions during testing better.

### Related Work

Several works employing ML and DL techniques have been present in the literature during recent years for the classification of colon and lung cancer subtypes from histopathological images from private and public (LC25000) datasets. These works are stratified into 3-class classification of lung cancer subtypes (adenocarcinoma, squamous cell carcinoma, and benign), 2-class classification of colon cancer subtypes (adenocarcinoma and benign), and 5-class classification of both lung and colon cancer subtypes, which are given in Table 1. In [21], a custom CNN model with heavy data augmentation from 298 microscopy images was developed and achieved an overall accuracy of 71.1% for subtyping lung cancer into adenocarcinoma, squamous cell carcinoma, and small cell carcinoma. In another recent study using LC25000 dataset [22], lung cancer subtyping is performed using a custom-made CNN, obtaining an accuracy of 97.2%. Furthermore, in [23], colon cancer subtyping was only implemented using a CNN and principal component analysis (PCA) from LC25000, and the framework has a classification accuracy of 99.8%. Few studies exist using feature extraction from the histopathology images and different ML classifiers, including random forest (RF) and XGBoost, for the lung and colon cancer subtyping and achieved accuracies above 96.3% [24,25].

A multi-input dual-stream capsule neural network was proposed [26] using LC25000 images that employed several pre-processing strategies, including color balancing, gamma correction, image sharpening and multi-scale fusion, to obtain an accuracy of 99.6%. Similarly, Ref. [27] employs histogram equalization as the pre-processing step followed by pretrained AlexNet to improve the colon cancer classification. In other recent studies, pretrained DarNet-19 and support vector machine classifier [28], DenseNet-121, and RF classifier [29] were developed and demonstrated 99.7% and 98.6% accuracy, respectively. Integration of deep feature extraction and ensemble learning with high-performance filtering was found to be effective in a recent work [30] with an accuracy of 99.3% using LC25000 data. Lastly, a custom CNN model from the same dataset followed by several dimensionality reduction methods, such as PCA, discrete Fourier transform, and fast Walsh-Hadamard transform, was employed to obtain 99.6% accuracy for the five-class classification [31].

Although some previous studies obtained accuracies above 99.5%, they lacked explainability and incorporated extensive pre-processing steps. Therefore, the present study aimed at using compound scaling-inspired *EffcientNetV2* models for the five-class classification with added interpretability using the gradCAM method. Eventually, our framework outperformed all the existing methods based on LC25000 dataset with an overall test accuracy of 99.98%.

**Table 1:** Previous works on classifying lung and colon cancer subtypes using different machine learning and deep neural network methods based on LC25000 dataset and a private dataset. CNN: convolution neural network. ML: machine learning, PCA: principal component analysis, DWT: discrete wavelet transforms, SVM: support vector machine, RF: random forest, BA: balanced accuracy, AUC: area under the receiver operating characteristic curve, MCC: Matthew’s correlation coefficient, FWHT: fast Walsh-Hadamard transform.

Study	Year	Method	Dataset	Interpretability	Performance (%)
Chehade A. H. et al. [25]	2022	ML classifiers	LC25000	No	Accuracy: 99.0 F1-score: 98.80
Masud M. et al. [24]	2021	ML classifiers	LC25000	No	Accuracy: 96.33
Ali M. et al. [26]	2021	Multi-input capsule neural network	LC25000	No	Accuracy: 99.58
Togacar M. [28]	2021	DarkNet-19 and SVM	LC25000	No	Accuracy: 99.69
Mehmood S. et al. [27]	2022	Image enhancement and AlexNet	LC25000	No	Accuracy: 98.40
Teramoto A. et al. [21]	2017	Custom CNN model	Private dataset (298 microscopic images)	No	Accuracy: 71.10 (Only lung cancer)
Attallah O. et al. [31]	2022	Custom CNN + PCA, FWHT, DWT	LC25000	No	Accuracy: 99.60
Hatuwal B. K. et al. [22]	2020	Custom CNN	LC25000	No	Accuracy: 97.20 (Only lung cancer)
Mangal S. et al. [32]	2020	Custom CNN	LC25000	No	Accuracy: 96.50
Talukder Md. A. et al. [30]	2022	Hybrid ensemble learning	LC25000	No	Accuracy: 99.30
Kumar N. et al. [29]	2022	DenseNet121 and RF	LC25000	No	Accuracy: 98.60 F1-score: 98.50
Hasan Md. I. et al. [23]	2022	Custom CNN and PCA	LC25000	No	Accuracy: 99.80 (Only colon cancer)
Present study	2023	*EffcientNetV2*	LC25000	Yes	Accuracy: 99.97 F1-score: 99.97 BA: 99.97 AUC: 99.99 MCC: 99.96

## 2. Methods

### 2.1. Dataset

For this study, we considered a publicly available dataset LC25000 [33]. Initially, 250 color images for each lung and colon cancer subtype were acquired using Leica microscope LM190 HD camera connected to an Olympus BX41 microscope, constituting 1250 images before data augmentation. Then, the 250 images for each cancer subtype were increased to 5000 by using augmentation methods, including right and left rotations and vertical and horizontal flips. Thus, after data augmentation, the dataset consists of 25,000 regular histopathology images. The original spatial resolution of the images was 1024 × 768, but they were cropped to 768 × 768 before applying the data augmentation. Eventually, for the current study, the spatial resolution of the images was changed to 224 × 224 by resizing.

**Figure 1:**
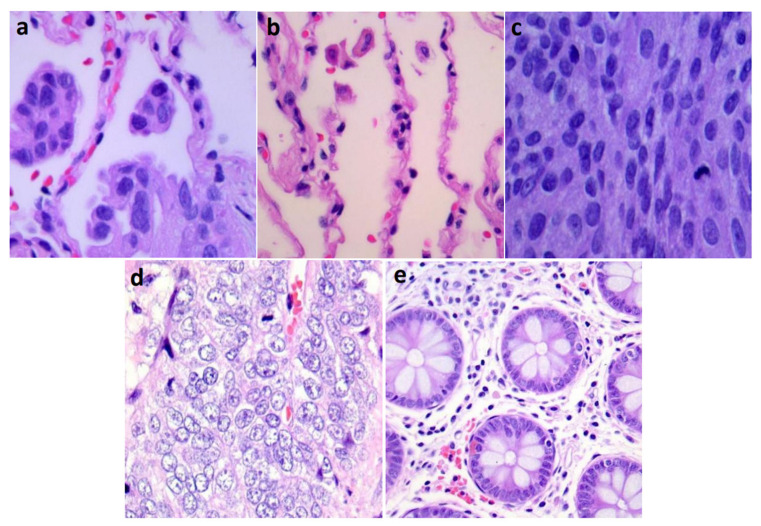
Sample lung and colon cancer histopathological images from LC25000 dataset. (**a**) lung-adenocarcinoma, (**b**) lung-benign, (**c**) lung-squamous cell carcinoma, (**d**) colon-adenocarcinoma, (**e**) colon-benign.

For a fair differentiation with existing literature, the percentage of data used in training, validation, and testing from LC25000 is considered to match with existing studies, i.e., 80% of the data was used for cross-validation and the remaining 20% for testing. The images in the dataset were labeled as follows: 0 for lung-adenocarcinoma, 1 for lung-benign, 2 for lung-squamous cell carcinoma, 3 for colon-adenocarcinoma, and 4 for colon-benign by experienced pathologists. Example histopathological images with lung and colon cancer subtypes are shown in Figure 1. Furthermore, the dataset stratified with respect to lung and colon cancer subtypes are given in Table 2 for the train, validation, and test sets.

### 2.2. Physiological Mechanims of Lung and Colon Cancers

In this subsection, we briefly described the pathophysiological mechanisms about the lung and colon cancer subtypes dealt in the present study. Lung adenocarcinoma and squamous cell carcinoma falls under the category of non-small cell lung cancers where squamous carcinoma frequently occurs as a central endobronchial lesion, and adenocarcinoma has a tendency to start in the lung periphery and invade the pleura [34]. Lung benign is non-cancerous and will not spread to the surrounding tissues. Most occurring lung benign include hamartomas that usually occurs in outer portion of lung connective tissue and bronchial adenomas that grow in the bronchi and in the ducts or mucus glands of the windpipe. Colon adenocarcinoma and benign occur in a pedunculated polyp, sessile polyp, or stricture. Polyp is an abnormal chunk of cells that also grow inside the colon. Small polyps rarely contain cancer [35].

### 2.3. EffcientNetV2 and Compound Scaling

**Figure 2:**
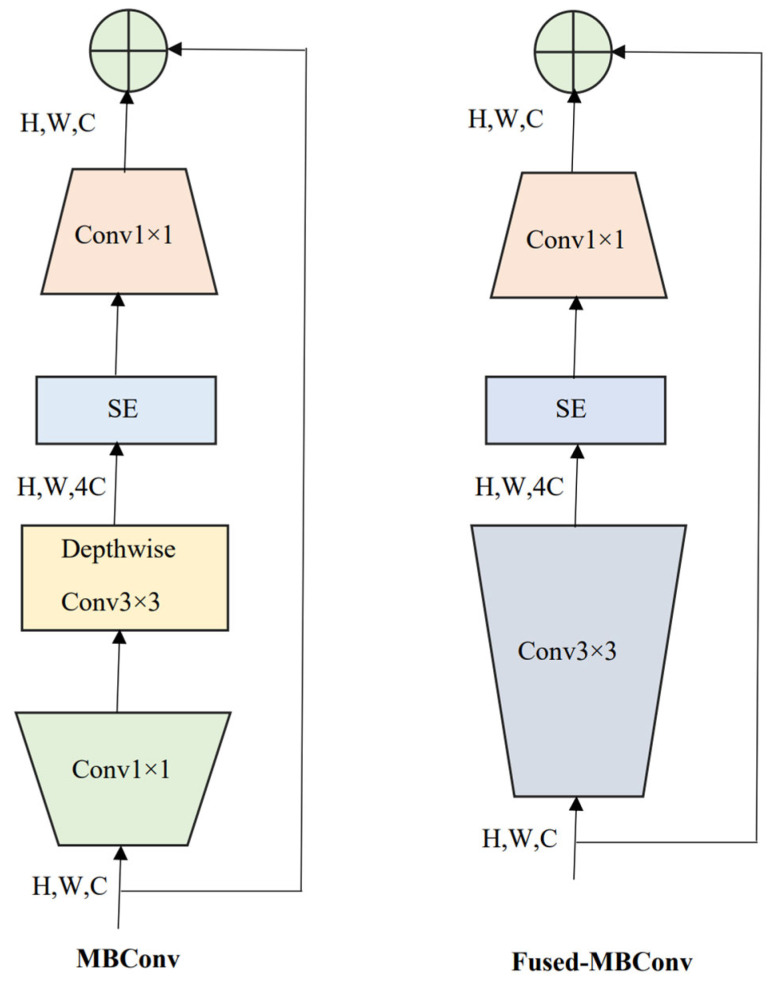
MBConv and Fused-MBConv layers are used as building blocks of *EffcientNetV2* models. SE: squeeze and excitation block. H, W, C: image height, width, and the number of channels.

*EffcientNetV2* [10], the next version of *EffcientNetV1* [9], is a novel family of deep CNNs focusing on two significant aspects: enhancing the training speed and parameter efficiency. To accomplish this task, a combination of training-aware NAS, and compound scaling were used. The faster training was achieved by employing both MBConv and Fused-MBConv layers. Here, MBConv layers are the basic building blocks of MobileNetV2 [36] constructed from the inverted residual blocks. To obtain the Fused-MBConv layer, the first two blocks (depth-wise 3 × 3 convolution and the expansion 1 × 1 convolution block) of MBConv were replaced by a regular 3 × 3 convolution block, as shown in Figure 2. Afterward, a squeeze and excitation block in both MBConv and Fused-MBConv layers was used to weigh different channels adaptively. Finally, a 1 × 1 squeeze layer was inserted to reduce the number of channels equal to the channels present in the input of either MBConv or Fused-MBConv layers.

In the present work, we considered *EffcientNetV2-L*, *-M,* and *-S* models that employed Fused-MBConv blocks in the initial layers. The *EffcientNetV2-S* model architecture begins with a standard 3 × 3 convolution layer followed by three Fused-MBConv and three MBConv blocks. The eventual layers contain a 1 × 1 convolution, pooling, and concluded by a fully connected layer. Furthermore, the *EffcientNetV2-S* model was scaled up using a compound scaling strategy to get *EffcientNetV2-M* and *-L* models. The idea behind compound scaling is to balance the dimensions of depth (*d*), width (*w*), and input image resolution (*r*) by scaling them to a constant ratio. Mathematically, it was formulated as given below.

(1) $$d=\alpha^{\varphi },\;w=\beta^{\varphi },\;r=\gamma^{\varphi }\;\mathrm{such}\;\mathrm{that}\;\alpha .\beta^{2}.\gamma^{2}=2$$

The values of α, β, and γ are always greater than or equal to one and could be determined by grid search. Intuitively, φ determines the extra computational resources required for model scaling, which is user defined. In practice, the convolution operations dominate the computational cost in CNNs. Hence, scaling a CNN using Equation (1) would roughly increase the floating-point operations per second (FLOPS) by ${\left(\alpha .\beta^{2}.\gamma^{2}\right)}^{\varphi }$. However, based on the constraint set in Equation (1), for any new φ, the FLOPS in total will approximately increase by $2^{\varphi }$. By progressively increasing the size of the image during training, the training speed was further improved. However, this gradual increase in the image size during training often leads to a drop in performance which was handled by adaptive regularization schemes, such as data augmentation and dropout. That means for smaller image sizes, weak augmentation was used and vice versa. Furthermore, for complete details, refer to [9].

### 2.4. Model Training and Validation

The final softmax layer of the pre-trained *EffcientNetV2-S*, *-M*, and *-L* models were discarded, and a new softmax layer is added to classify lung and colon cancer subtypes. The model cross-validation and testing were conducted under the Google Colab Pro cloud environment using TensorFlow 2.0 with high-level *Keras* API at the backend. Furthermore, all the hyperparameters for all the models were empirically selected, and hence the validation set was used to ensure that the individual models were not over-fitting during training. For training, the *Adadelta* optimizer was used at 0.1 learning rate, 32 batch size, and 5 epochs. Since it is a five-class classification problem, sparse categorical cross-entropy (SCCE), as given in Equation (2), was used as the loss function.

(2) $$SCCE_{loss}=-\frac{1}{N}\overset{N}{\underset{i=0}{\sum }}\overset{5}{\underset{j=1}{\sum }}y_{j}log({\hat{y}}_{j})$$

Above, *N* is the total number of images during training/validation, ${\hat{y}}_{j}$ is the label of predicted class, and $y_{j}$ is the label of the true class. For all three models, the parameters of the last 50 percent of layers were fine-tuned during training, and the parameters of the first half of the network remain unaltered. We have used two repetitions for splitting the data into training, and testing and the average performance metric values are reported.

### 2.5. Visual Saliency Maps

To better understand the model’s decisions on where it is keeping more attention on the histopathology image during prediction, the visual saliency maps are created for each *EffcientNetV2* model using gradCAM [19] for all lung and colon cancer subtypes. To obtain the gradCAM map $L_{gradCAM}^{c}\in {\mathbb{R}}^{u\times v}$ of width *u* and height *v* for class *c*, indicating the most representative regions, we initially compute the first order derivative of the score for class *c* denoted as $y^{c}$ (before the softmax), with respect to the feature maps $A^{k}$ of the last convolutional layer. Furthermore, these first order derivatives propagated back are global mean pooled over the width and height of $A^{k}$ (indexed by *i* and *j*, respectively) to get the neuron significance weights $\alpha_{k}^{c}$. Mathematically, it is described as given below in Equation (3). Here, Z is the product of the width and height of the feature map $A^{k}$. The importance weights $\alpha_{k}^{c}$ captures the ‘importance’ of feature map $A^{k}$ for a class of interest *c*. Furthermore, to get $L_{gradCAM}^{c}$, a weighted sum of final convolution layer output maps followed by ReLU (rectified linear unit) is performed as shown in Equation (4). Furthermore, ReLU is given in Equation (5). A ReLU is applied evatually to extract the ‘positive’ features that influence the class of interest.

(3) $$\alpha_{k}^{c}=\frac{1}{Z}\underset{i}{\sum }\underset{j}{\sum }\frac{\partial y^{c}}{\partial A_{ij}^{k}}$$

(4) $$L_{gradCAM}^{c}=ReLU\left(\underset{k}{\sum }\alpha_{k}^{c}A^{k}\right)$$

(5) $$ReLU\left(x\right)=\{\begin{array}{c} x,\;\;x>0\\ 0,\;\;x\leq 0 \end{array}$$

### 2.6. Evaluation Metrics

To conduct the performance evaluation of the proposed models, the Python-based scikit-learn toolbox was used. The metrics include accuracy, F1-score, balanced accuracy (*BA*), area under the receiver operating characteristic curve (AUC), and Matthew’s correlation coefficient (MCC), as described in the below equations. Here, F1-score is calculated from the harmonic mean of precision and sensitivity, whereas *BA* is computed as the average of recall and specificity. Since it was a five-class classification study, the performance scores are obtained from the corresponding confusion matrix (*CM*) by employing one vs. rest approach. Given a specific class, the correctly classified images are categorized as true positives (*TP*). The false positives (*FP*) are the misclassifications above the half-diagonal of *CM*. The number of correctly classified present in the diagonal of CM other than the specific class are called true negatives (*TN*). Eventually, the misclassifications below the half diagonal are considered as false negatives (*FN*).

(6) $$accuracy=\frac{TP+TN}{TP+TN+FP+FN}$$

(7) $$F1-score=\frac{2*precision*recall}{precison+recall}$$

(8) $$BA=\frac{sensitivity+specificity}{2}$$

(9) $$sensitivity\;\left(recall\right)=\frac{TP}{TP+FN}$$

(10) $$specificity=\frac{TN}{TN+FP}$$

(11) $$precision=\frac{TP}{TP+FP}$$

(12) $$MCC=\;\frac{TP.TN-FP.FN}{\sqrt{\left(TP+FP\right)\left(TP+FN\right)\left(TN+FP\right)\left(TN+FN\right)}}$$

## 3. Results

**Figure 3:**
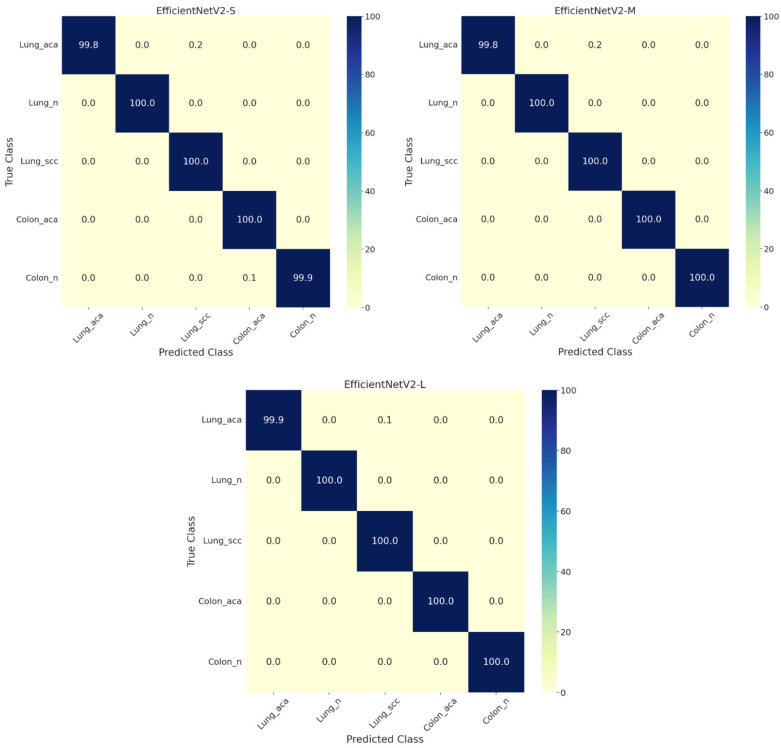
Multi-class confusion matrices for the test set for lung and colon cancer classification by employing *EffcientNetV2-S*, *-M*, and *-L* models. Lung_aca: lung adenocarcinoma, Lung_n: lung benign, Lung_scc: lung squamous cell carcinoma, Colon_aca: colon adenocarcinoma, Colon_n: colon benign.

All the models converged within five iterations during the cross-validation. Therefore, the evaluation scores during validation are very close to the performance scores during testing. Table 3 presents the complete evaluation details of the proposed *EffcientNetV2-S*, *-M*, *-L* models on the test set. The *EffcientNetV2-L* model performed better among all three, with an accuracy of 99.97% and an AUC of 99.99%. However, the other two models (small and medium) abilities are also very close to the performance metrics of the large model. For example, from Figure 3, we can see that the large model achieved almost 100% accuracy for the three-class stratification of lung cancer. Similarly, the medium model has obtained 100% accuracy for the two-class classification of colon cancer.

**Figure 4:**
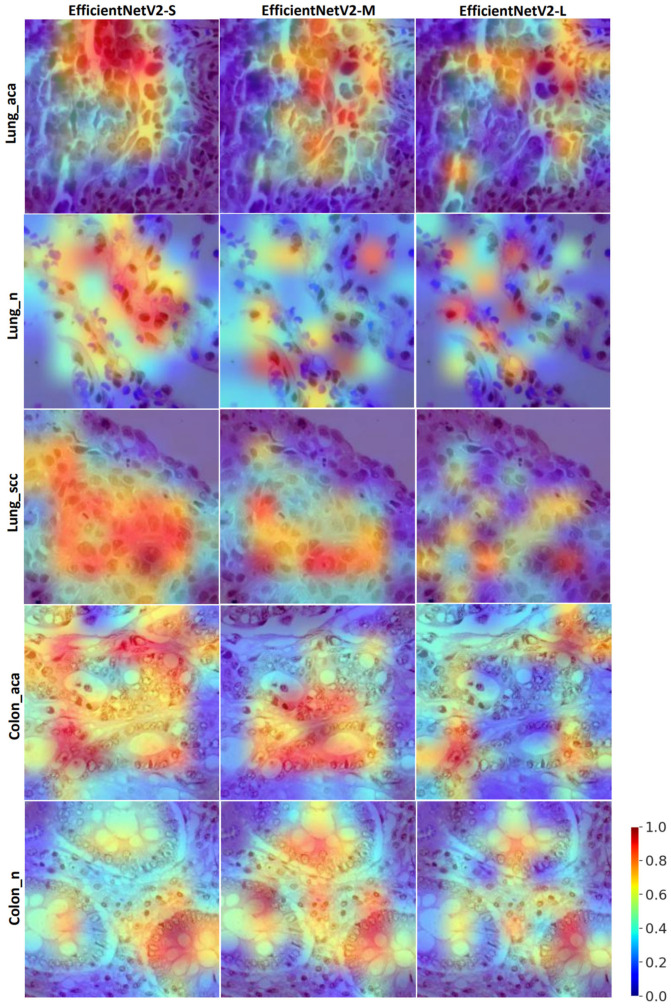
Visual saliency maps for explainability of the model’s decisions during class prediction, created using gradCAM. For each class, one image is randomly picked from the test set. Lung_aca: lung adenocarcinoma, Lung_n: lung benign, Lung_scc: lung squamous cell carcinoma, Colon_aca: colon adenocarcinoma, Colon_n: colon benign. The red color in the maps indicates that more attention is given in those regions, and the blue color indicates that less attention is put to those regions during model prediction.

Furthermore, Figure 4 shows the visual saliency maps for a sample image for all lung and colon cancer subtypes using gradCAM. For comparison, the maps were generated for all three employed models of *EffcientNetV2*. In general, the highlighted regions in the histopathology image are similar among the different models. However, some notable differences are present. For instance, the most activated regions during colon adenocarcinoma prediction using medium and large models are slightly different. In addition, the activated regions for the three-class lung cancer classification are wider for the small model compared to the medium and large models. Furthermore, we have given a color bar applicable to all the sub-saliency maps present in Figure 4 for quantitative estimate of attention. Here, red color indicates more attention (maximum value being one), and blue color indicates less attention (minimum value being zero) that the model put over the test histopathology image during the class prediction.

## 4. Discussion

In the present study, we proposed a pipeline using pretrained *EffcientNetV2* models (*L*, *M*, and *S*) for the automated classification of lung and colon cancer subtypes from histopathology images of LC25000 dataset. These compound-driven architectures outperformed the existing works on the same dataset by achieving an accuracy up to 99.97%, including all five classes indicating the power of both compound scaling and TL. Hence, the models may essentially replace the pathologist and make the classification of lung and colon cancer fully automatic. Furthermore, our framework is end-to-end, requiring neither any pre-processing methods nor any dimensionality reduction strategies as employed in some previous studies to achieve accuracies above 99.5% [26,31]. For instance, the method in [27] used histogram equalization for colon cancer images to boost the overall accuracy from 89% to 98.4%, and we believe that extensive pre-processing may hamper the generalizable ability of the model to unseen data. The better overall performance of *EffcientNetV2-L* model could be due to the presence of a greater number of MBConv and Fused-MBConv layers that helped in learning the most relevant abstract features required for very accurate classification.

Looking at the visual saliency maps in Figure 4, we can understand the most activated regions during the target class prediction by the models. In general, the most activated areas in the image are widespread for small model relative to medium and large models since the small model has comparatively few parameters/layers, and to achieve better differentiability among subtypes, attention over large area of the image may be necessary. This trend was more apparent for lung cancer subtypes and colon adenocarcinoma. Interestingly, the medium model demonstrated wider activations for colon benign compared to the small and large models. Overall, we can observe from the saliency maps that all models’ feature abstraction is from the appropriate areas of the histopathological images consistent across all lung and colon cancer subtypes. Furthermore, the color bar guides the pathologists to quantitively measure (in the scale between zero and one) the amount of attention/importance the model put over the subregions of the test histopathology images during their class prediction. Furthermore, these visual saliency maps may assist pathologists in potentially designing individual treatment strategies.

Since the dataset was largely generated by augmenting the original dataset containing 250 histopathology images for each cancer subtype, data augmentation may not provide true data variability. Hence, future studies should involve testing the proposed models on larger datasets created without using any data augmentation. Although the hyperparameters during training were chosen empirically, a thorough grid search, including the selection of optimizer, could be conducted using cross validation. Nonetheless, the performance metrics are quite impressive on the test set across all three models, thus, strongly supporting the empirically chosen hyperparameters. In addition, it will be interesting to implement few-shot learning methods [37] that work based on small sample sizes as an alternative to increasing the dataset size using heavy data augmentation.

Deep learning with *EffcientNetV2* large, medium, and small models with high accuracies of 99.96% can perform an important role in diagnosis and treatment of carcinoma lung and colon. This algorithm can be employed to analyze the vast amounts of data generated for cancer diagnosis, including images of tissue samples viewed under a microscope, genetic data, and other clinical information. One of the key advantages of this deep learning model is its ability to analyze large datasets and identify patterns that may be difficult for human experts to discern. This can significantly improve the accuracy of cancer diagnosis, particularly in cases where subtle differences between healthy and cancerous tissue may be difficult to distinguish. In addition to improving diagnosis, it can also be used to develop personalized treatment plans for cancer patients according to disease severity. This can be done by analyzing data from large numbers of patients with similar genetic profiles. The presented algorithm can identify the most effective treatment options for individual patients based on their unique characteristics. Overall, the role of deep learning in CA lung and CA colon histopathology is significant, as it has the potential to improve the accuracy of cancer diagnosis, reduces histopathologist’s burden, identify new treatment options, and ultimately help save lives.

## 5. Conclusions

The *EffcientNetV2* based -*L*, -*M*, and -*S* models presented in this study have achieved accuracies above 99.96% AUCs of 99.99%, and MCC of up to 99.96% on the test dataset for the five-class classification that includes three lung cancer subtypes and two colon cancer subtypes from histopathology images. The performance is superior to the existing works using the LC25000 dataset, and, furthermore, we employed gradCAM to highlight the most important regions while target class prediction. The performance metrics of the classification are marginally superior for -*M* and -*L* models compared to -*S* model. Hence, the proposed framework may assist pathologists in fully automating the lung and colon cancer subtyping from histopathological images and interpretability. In the future, we would like to propose lightweight models for the same task that could be deployable on edge devices. The code of the proposed pipeline can be found here.

## Figures and Tables

**Table 2:** Number of lung and colon cancer histopathology images in the training, validation, and testing. aca: adenocarcinoma, n: benign, scc: squamous cell carcinoma.

	Lung-aca	Lung-n	Lung-scc	Colon-aca	Colon-n
Training	3600	3600	3600	3600	3600
Validation	400	400	400	400	400
Testing	1000	1000	1000	1000	1000

**Table 3:** Evaluation metrics on the test set for classifying lung and colon cancer subtypes using *EffcientNetV2*-*S/M/L* models given in percentages. BA: balanced accuracy, AUC: area under the curve, MCC: Matthew’s correlation coefficient.

	*EffcientNetV2*-S	*EffcientNetV2*-M	*EffcientNetV2*-L
Accuracy	99.90	99.96	99.97
AUC	99.99	99.99	99.99
F1-Score	99.90	99.96	99.97
BA	99.90	99.97	99.97
MCC	99.87	99.94	99.96

## Data Availability

The data used in the study is publicly available from Kaggle.
